# Complementary Modular Microcircuits of the Rat Medial Entorhinal Cortex

**DOI:** 10.3389/fnsys.2017.00020

**Published:** 2017-04-10

**Authors:** Saikat Ray, Andrea Burgalossi, Michael Brecht, Robert K. Naumann

**Affiliations:** ^1^Bernstein Center for Computational Neuroscience, Humboldt University of BerlinBerlin, Germany; ^2^Werner-Reichardt Centre for Integrative NeuroscienceTübingen, Germany; ^3^German Center for Neurodegenerative DiseasesBerlin, Germany; ^4^Max-Planck-Institute for Brain ResearchFrankfurt, Germany

**Keywords:** calbindin, zinc, acetylcholinesterase, modularity, medial entorhinal cortex, presubiculum, parasubiculum, mMEC

## Abstract

The parahippocampal region is organized into different areas, with the medial entorhinal cortex (MEC), presubiculum and parasubiculum prominent in spatial memory. Here, we also describe a region at the extremity of the MEC and bordering the subicular complex, the medial-most part of the entorhinal cortex. While the subdivisions of hippocampus proper form more or less continuous cell sheets, the superficial layers of the parahippocampal region have a distinct modular architecture. We investigate the spatial distribution, laminar position, and putative connectivity of zinc-positive modules in layer 2 of the MEC of rats and relate them to the calbindin-positive patches previously described in the entorhinal cortex. We found that the zinc-positive modules are complementary to the previously described calbindin-positive patches. We also found that inputs from the presubiculum are directed toward the zinc-positive modules while the calbindin-positive patches received inputs from the parasubiculum. Notably, the dendrites of neurons from layers 3 and 5, positive for Purkinje Cell Protein 4 expression, overlap with the zinc modules. Our data thus indicate that these two complementary modular systems, the calbindin patches and zinc modules, are part of parallel information streams in the hippocampal formation.

## Introduction

One of the most important functions which the brain performs, is the ability to remember. The importance of the hippocampal and parahippocampal regions in memory arose with the case of Henry Molaison or HM (Scoville and Milner, 1957) which established a direct link between these areas and memory. Further, the discovery of place cells in the hippocampus (O’Keefe and Dostrovsky, 1971) opened a new perspective for understanding cognition (Nadel and O’Keefe, 1974; O’Keefe and Nadel, 1978) by providing a cellular basis for spatial navigation.

The medial entorhinal cortex (MEC) has recently emerged as a central hub in the circuits for spatial memory through the discovery of grid cells (Hafting et al., 2005). These are neurons which increase their activity in certain locations, tiling space in a hexagonal grid pattern. The MEC also contains a host of other cells which are spatially modulated including border cells (Solstad et al., 2008) and head direction cells (Sargolini et al., 2006). Layer 2 of the MEC contains the largest density of pure grid cells (Hafting et al., 2005; Boccara et al., 2010) and also contains other spatial cells like border and head direction cells.

In layer 2 of MEC there are two distinct principal cell types based on electrophysiological properties: intrinsically rhythmic neurons and neurons that do not show intrinsic rhythmicity (Alonso and Llinas, 1989; Alonso and Klink, 1993). A number of recent studies have also identified molecular markers that correspond to a large extent to the morphological and physiological neuron types (Varga et al., 2010; Kitamura et al., 2014; Ray et al., 2014; Tang et al., 2014). Specifically, Varga et al. (2010) demonstrated that reelin-positive cells show electrophysiological parameters traditionally assigned to stellate cells whereas electrophysiological properties of calbindin positive cells are similar to those of pyramidal cells. Ray et al. (2014) and Tang et al. (2014) found that calbindin positive and negative cells displayed distinct temporal firing patterns *in vivo*, with calbindin-positive pyramidal neurons displaying stronger spike rhythmicity in the theta frequency range (4–12 Hz). In addition, Wolframin (Wfs-1), which is expressed in modular structures in the entorhinal cortex and the parasubiculum (Ray and Brecht, 2016), was shown to be a marker for pyramidal cells and largely overlaps with calbindin expression in the MEC (Kitamura et al., 2014).

A number of histochemical markers are distributed in a modular pattern in rodent MEC, yet their relation to the principal cell distribution is largely unknown. In Ray et al. (2014) we demonstrated that acetylcholinesterase patches (Mathisen and Blackstad, 1964; Slomianka and Geneser, 1991) overlap with patches of calbindin-positive cells. Other markers revealing modularity include cytochrome oxidase (Burgalossi et al., 2011) and zinc ions (Slomianka, 1992; Slomianka and Geneser, 1997). In addition, a number of afferent fiber systems such as commissural fibers (Blackstad, 1956), inputs from presubiculum and parasubiculum (Köhler, 1985), and intrinsic connections originating in layer 5 of MEC (Köhler, 1986b) display a modular pattern of termination in MEC. Injections of anterograde tracers into the presubiculum were shown to label a band-like topographically organized pattern in the MEC (Honda and Ishizuka, 2004) that may encompass a number of smaller scale modules (Köhler, 1985). This band-like topographic organization of projections is conserved and propagated throughout the entire hippocampal region (Honda et al., 2012; Honda and Ishizuka, 2015). The spatial activity pattern of individual grid cells is also modularly organized, along the dorso-ventral axis in MEC (Barry et al., 2007; Brun et al., 2008). There is, however, no consensus as to how and if at all physiologically identified modules relate to anatomical modules in the MEC.

The hippocampal formation is among the most intensely studied parts of the brain (Lorente de Nó, 1933, 1934; Blackstad, 1956; Haug, 1973; Slomianka, 1992). It consists of two major parts (**Table 1**): a core region (dentate gyrus, mossy cells, CA fields, and subiculum) and a surrounding belt or parahippocampal region (presubiculum, parasubiculum, medial and lateral entorhinal cortex, post-rhinal and perirhinal cortex, and, possibly, medial parts of retrosplenial cortex) (Lorente de Nó, 1933, 1934; Blackstad, 1956; Köhler, 1986a; Slomianka and Geneser, 1991; Amaral and Lavenex, 2009; Cappaert et al., 2015). Yet, considerable difficulties remain in identifying subdivisions of this region, especially when considering dorso-ventral differences (Dong et al., 2009; Fanselow and Dong, 2010; Cembrowski et al., 2016; Ishihara and Fukuda, 2016) or cross-species comparisons (Ding, 2013). There is a need to consider both, a broader evolutionary perspective of the medial pallium, i.e., the hippocampal formation (Insausti, 1993; Slomianka et al., 2012; Abellán et al., 2014; Naumann et al., 2016; Striedter, 2016) and more detailed studies of specific circuits in a few “model species.” Here, we focus on the latter and aim at providing a map of modular structures in the rat MEC and their relation with neighboring areas in the parahippocampal region, as a basis for investigations of the structural determinants of hippocampal circuit function.

**Table 1:** Regions in the rat hippocampal formation.

Region name	Abbreviation
Dentate gyrus	DG
Cornu ammonis regions	CA1, CA2, CA3
Subiculum	Sub
Presubiculum	PrS
Parasubiculum	PaS
Medial entorhinal cortex	MEC
Medial-most medial entorhinal cortex	mMEC
Lateral entorhinal cortex	LEC
Retrosplenial granular cortex	Rsg
Retrosplenial agranular cortex	Rsa
Post-rhinal cortex	PoR

## Materials and Methods

All experimental procedures were performed according to German guidelines on animal welfare.

### Animals

Male and female adult wistar and long evans rats (*N* = 58) were used in the study. All experimental procedures were performed according to the German guidelines on animal welfare and approved by the local institution in charge of experiments using animals (Landesamt für Gesundheit und Soziales Berlin, permit number T0106/14 and T0078/16; Regierungspraesidium Tuebingen, permit number CIN5/14).

### Tissue Preparation

Animals were anesthetized by isoflurane, and then euthanized by an intraperitoneal injection of 20% urethane or overdose of pentobarbital. They were then normally perfused transcardially with first 0.9% phosphate buffered saline solution, followed by 4% formaldehyde, from paraformaldehyde, in 0.1 M phosphate buffer (PFA). For zinc histochemistry, there was an additional perfusion of a sodium sulfide solution prior to perfusing with PFA. After perfusion, brains were removed from the skull and post-fixed in PFA overnight. Brains were then transferred to 10% sucrose solution for one night and subsequently immersed in 30% sucrose solution for at least one night for cryoprotection. The brains were embedded in Jung Tissue Freezing Medium (Leica Microsystems Nussloch, Germany), and subsequently mounted on the freezing microtome (Leica 2035 Biocut) to obtain 20–60 μm thick horizontal, sagittal, or tangential sections parallel to the pia.

Tangential sections of the MEC were obtained by separating the entorhinal cortex from the remaining hemisphere by a cut parallel to the surface of the MEC. For subsequent sectioning the surface of the entorhinal cortex was attached to the block face of the microtome.

### Histochemistry

#### Acetylcholinesterase Activity

Acetylcholinesterase (AChE) was stained following the method of Tsuji (1998) and Ichinohe et al. (2008). After washing in a mixture containing 1 ml of 0.1 M citrate buffer (pH 6.2) and 9 ml 0.9% saline (CS), sections were incubated with CS containing 3 mM CuSO_4_, 0.5 mM K_3_Fe(CN)_6_, and 1.8 mM acetylthiocholine iodide for 30 min. After rinsing in PB, sections were intensified in PB containing 0.05% 3,3′- Diaminobenzidine (DAB) and 0.03% nickel ammonium sulfate.

#### Zinc Activity

For the visualization of synaptic zinc, sections were developed as described by Danscher (1981). In brief, sections were exposed to a solution containing gum arabic, citrate buffer, hydroquinone, and silver lactate for 60–120 min, in the dark at room temperature. Development of reaction products was checked under a microscope and terminated by rinsing the sections in 0.01 M PB and, subsequently, several times in 0.1 M PB (Ichinohe and Rockland, 2004).

#### Immunohistochemistry

Tangential, horizontal, and sagittal sections were immunostained with the antibodies listed in **Table 2**. For multiple antibody labeling, antibodies raised in different host species were combined. In each series of sections the primary antibody was omitted in one section to control for secondary antibody specificity. This always led to complete absence of staining.

**Table 2:** Antibodies.

Name	Species	Cat.	RRID	Source	Supplier	Dilution	Specificity
Calbindin D-28k	Mouse mono- clonal	300	AB_10000347	Purified from chicken gut	Swant	1:5000	Celio et al., 1990
Calbindin D-28k	Rabbit poly- clonal	CB38	AB_10000340	Purified from recombinant rat calbindin D-28k	Swant	1:5000	Airaksinen et al., 1997
Calretinin	Mouse mono-clonal	6B3	AB_10000320	Purified using recombinant human calretinin-22k	Swant	1:5000	Zimmermann and Schwaller, 2002
Chloecsytokinin	Goat poly-clonal	Sc-21617	AB_2072464	Purified using peptide mapping near C-terminus of CCK of human origin	Santa Cruz	1:500	Takahashi et al., 1985
Parvalbumin	Mouse mono-clonal	PV235	AB_10000343	Purified from carp muscles	Swant	1:5000	Celio and Heizmann, 1981
Parvalbumin	Goat poly-clonal	PVG213	AB_10000345	Purified from rat muscle parvalbumin	Swant	1:5000	Schwaller et al., 1999
Purkinje cell protein 4	Rabbit poly-clonal	HPA005792	AB_1855086	Purified using protein epitope signature tag	Sigma-aldrich	1:1000	Sangameswaran et al., 1989
Wolframin syndrome 1	Rabbit poly-clonal	11558-1-AP	AB_2216046	Antigen affinity purification	Proteintech	1:500	Inoue et al., 1998

#### Calbindin

The mouse monoclonal anti-calbindin (CB) antibody was raised using hybridization of mouse myeloma cells with spleen cells from mice immunized with the CB D-28k that was purified from the chicken gut (Celio et al., 1990). This monoclonal antibody is not known to cross-react with other known calcium binding-proteins and specifically stains the 45Ca-binding spot of CB D-28k (MW 28,000, IEP 4.8) of different mammals in a two-dimensional gel (manufacturer’s technical information). The rabbit polyclonal anti-CB antiserum was raised against recombinant rat calbindin D-28k (Airaksinen et al., 1997). It cross-reacts with calbindin D-28k from many mammalian species. In immunoblots it recognizes a single band of approximately 27–28 kDa (manufacturer’s technical information).

#### Calretinin

The antibody against calretinin was produced in mice by immunization with recombinant human calretinin-22k (Zimmermann and Schwaller, 2002). Calretinin 22k is an alternative splice product of the calretinin gene and identical with calretinin up to Arg178. After fusion, hybridoma cells were screened with human recombinant calretinin as target and the clone 6B3 was selected. The antibody 6B3 recognizes an epitope within the first four EF-hands domains common to both calretinin and calretinin-22k (Zimmermann and Schwaller, 2002). It was evaluated for specificity and potency: (a) by Biotin–Avidin labeling of cryostate-, vibratome-, and paraffin-sections of 4% paraformaldehyde (or 10% buffered formalin) fixed brains (b) by immunoenzymatic labeling of immunoblots (c) by immunohistochemistry on tissue of calretinin knock-out mice. The product is a monoclonal antibody against calretinin a calcium-binding protein of the EFhand family related to calbindin D-28k and calmodulin (Rogers, 1987). The antibody reacts specifically with calretinin in tissue originating from human and rat. This antibody does not cross-react with calbindin D-28k or other known calcium binding-proteins, as determined by its distribution in the brain, as well as by immunoblots (manufacturer’s technical information).

#### Chloecystokinin

Cholecystokinin (CCK) is a 115 amino acid secreted protein belonging to the gastrin/cholecystokinin family. CCK has been shown to stimulate the growth of pancreatic cancer. As a peptide hormone, CCK induces gallbladder contraction and the release of pancreatic enzymes in the gut. Binding of CCK to CCK-A receptors stimulates amylase release from the pancreas, while binding to CCK-B receptors stimulates gastric acid secretion. The function of CCK in the brain is not clear. The CCK precursor is cleaved by proteases to produce a number of active cholecystokinins including CCK58, CCK58 desnonopeptide, CCK39, CCK33, CCK25, CCK18, CCK12, CCK8, CCK7, and CCK5. The gene encoding CCK maps to human chromosome 3p22.1 and mouse chromosome 9 F4 (manufacturer’s technical information).

#### Parvalbumin

The monoclonal anti-Parvalbumin is a mouse IgG1 produced by hybridization of mouse myeloma cells with spleen cells from mice immunized with parvalbumin purified from carp muscles. The antibody was evaluated for specificity and potency: (a) by indirect immunofluorescent or immunoperoxidase labeling as well as Avidin–Biotin staining of cryostat or vibratome-sections of 4% paraformaldehyde fixed tissue; (b) by immunoenzymatic labeling of immunoblots; (c) by radioimmunoassay (RIA). The product is a monoclonal antibody (McAB) against parvalbumin, a calcium-binding protein of the EF-hand family related to calmodulin and troponin-C. The antibody reacts specifically with parvalbumin in tissue originating from human, monkey, rabbit, rat, mouse chicken, and fish. The McAB specifically stains the 45Ca-binding spot of parvalbumin (MW 12′000 and IEF 4.9) in a two-dimensional “immunoblot.” In a RIA set up the McAB measures parvalbumin with a sensitivity of 10 ng/assay and an affinity of 7.9 × 1012 L/M.

The goat anti-parvalbumin was produced against rat muscle parvalbumin. It cross-react with some other species, including human parvalbumin. It can be used for immunoblotting and immunohistochemistry and does not stain the brain of parvalbumin knockout mice (manufacturer’s technical information).

#### Purkinje Cell Protein 4

The rabbit polyclonal anti-purkinje cell protein 4 (PCP4) antibody was raised by affinity purification using the recombinant protein epitope signature tag (PrEST) antigen (GAGATNGKDKTSGENDGQKKVQEEFDIDMDAPETERAAVAIQSQFRKFQKKK) as the affinity ligand. The specific reactivity against target PrEST antigen was validated on a protein array with 384 randomly selected antigens (manufacturer’s technical information).

#### Wolframin Syndrome 1

Wolfram syndrome protein (WFS1), also called wolframin, is a transmembrane protein, which is located primarily in the endoplasmic reticulum and its expression is induced in response to ER stress, partially through transcriptional activation. ER localization suggests that WFS1 protein has physiological functions in membrane trafficking, secretion, processing, and/or regulation of ER calcium homeostasis. It is ubiquitously expressed with highest levels in brain, pancreas, heart, and insulinoma beta-cell lines. Mutations of the WFS1 gene are responsible for two hereditary diseases, autosomal recessive Wolfram syndrome and autosomal dominant low frequency sensorineural hearing loss (manufacturer’s technical information).

#### Light and Fluorescence Microscopy

An Olympus BX51 microscope (Olympus, Shinjuku, Tokyo, Japan) was used to view the images using bright field microscopy. The microscope was equipped with a motorized stage (LUDL Electronics, Hawthorne, NY, USA) and a z-encoder (Heidenhain, Shaumburg, IL, USA). Images were captured using a MBF CX9000 (Optronics, Goleta, CA, USA) camera using Neurolucida or StereoInvestigator (MBF Bioscience, Williston, VT, USA).

A Leica DM5500B epifluorescence microscope with a Leica DFC345 FX camera (Leica Microsystems, Mannheim, Germany) was used to image the immunofluorescent sections. Alexa fluorophores were excited using the appropriate filters (Alexa 488 – L5, Alexa 546 – N3). The fluorescent images were acquired in monochrome and color maps were applied to the images post-acquisition. *Post hoc* linear brightness and contrast adjustment were applied uniformly to the image under analysis.

#### Anterograde Neuronal Labeling

Anterograde tracer solutions containing biotinylated dextrane amine (BDA) (10% w/v; 10,000 molecular weight) were injected in juvenile rats (∼150 g) under ketamine/xylazine anesthesia. Briefly, a small craniotomy was opened above the parasubiculum/presubiculum. Before injection, the pre- or parasubiculum was localized by electrophysiological recordings, based on cortical depth, characteristic signatures of the local field potential theta oscillations, and neuronal spiking activity. Glass electrodes with a tip diameter of 10–20 μm, filled with BDA solution, were then lowered into the target region. Tracers were either pressure-injected (10 injections using positive pressure of 20 psi, 10–15 s injection duration) or iontophoretically injected (7 s on/off current pulses of 1–5 mA for 15 min). After the injections, the pipettes were left in place for several minutes and slowly retracted. The craniotomies were closed by application of silicone and dental cement. The animals survived for 3–7 days before being transcardially perfused.

## Results

### Anatomical Organization of the Rat Parahippocampal Regions

**FIGURE 1:**
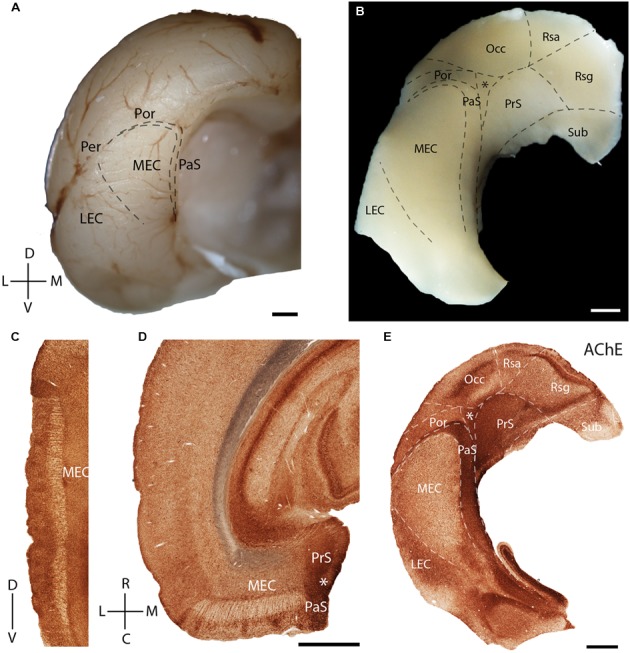
**Rat parahippocampal region**. Overview of the rat parahippocampal region in sagittal, horizontal, and tangential sections. **(A)** Posterior view of the left cortical hemisphere of a rat brain indicating the locations of medial entorhinal cortex (MEC), lateral entorhinal cortex (LEC), parasubiculum (PaS), perirhinal cortex (Per), and post-rhinal cortex (Por). **(B)** Brain slice prepared by a tangential cut through entorhinal cortex and adjacent regions, unfolded and gently flattened showing the positions of the subiculum (Sub), retrosplenial granular cortex (Rsg), retrosplenial agranular cortex (Rsa), occipital cortex (Occ) and the triangular region (^∗^) in addition to the MEC, LEC, PaS, Per, and Por. Sections stained for acetylcholinesterase activity (AChE) indicating the parahippocampal regions in a saggital **(C)**, horitzontal **(D)**, and tangential **(E)** section. Scale bars = 1 mm. D, dorsal; V, ventral; L, lateral; M, medial; R, rostral; C, caudal. Orientation in **(A)** also applicable to **(B,E)**. © 2014 The American Association for the Advancement of Science, **(A)** is adapted from Ray et al. (2014). Reprinted with permission from AAAS.

In the present study, we first describe the anatomical location and organization the MEC within the parahippocampal region. The MEC is located at the posterior pole of the rat cerebral cortex (**Figure 1A**, adapted from Ray et al., 2014). A tangential view of the parahippocampal region shows the position of the MEC, which is flanked rostromedially by the presubiculum (PrS) and parasubiculum (PaS). They, together with the post-rhinal cortex (Por) and occipital cortex (Occ) enclose a triangular region (marked by an asterisk in **Figure 1B**). The most dorsal part of the tangential section also shows parts of the subiculum (Sub) and retrosplenial cortex [retrosplenial granular cortex (Rsg); retrosplenial agranular cortex (Rsa)].

Acetylcholinesterase activity (**Figures 1C–E**) can be used to delineate chemoarchitectonic characteristics of the entorhinal cortex and neighboring regions (Mathisen and Blackstad, 1964). Most investigations of the rodent entorhinal region have used sagittal (**Figure 1C**) or horizontal (**Figure 1D**) sections. Variably sized islands of AChE staining are present in layer 2 of MEC (**Figures 1C,D**). To better resolve the topography of modular structures in the entorhinal cortex we use tangential sections of the entorhinal region as shown in **Figure 1E** (see Ray and Brecht, 2016 for details on preparation), an approach which has been widely used in sensory cortices (Woolsey and Van der Loos, 1970). Here and in the following figures, we always orient the section along the dorso-ventral axis, with the long axis of the PaS being vertically represented. A section through layer 3 of MEC cut from the tangential preparation in **Figure 1B** is shown in **Figure 1E**. Here, staining for AChE activity reveals clear architectonic borders of several cortical areas (Eckenstein et al., 1988), like that of the MEC with LEC and PaS (**Figure 1E**).

**FIGURE 2:**
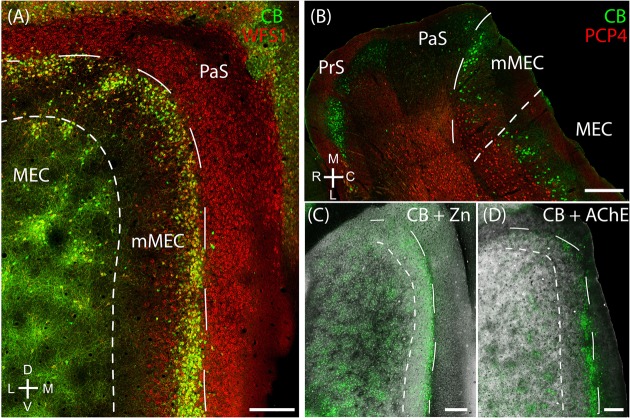
**Medial-most part of the medial entorhinal cortex (mMEC), a transitional region between medial entorhinal cortex and parasubiculum**. The region between the parasubiculum and calbindin patch region in the MEC is markedly different from the rest of the MEC and is termed as the medial-most MEC. **(A)** The mMEC is sandwiched between the MEC and PaS (red, Wfs1) on the medial end, and lacks calbindin patches (green) as illustrated in the tangential section. **(B)** A horizontal section shows that the mMEC, like the PaS extends from layer 1 to layer 3 of the MEC. **(C)** Same section processed for calbindin (green) and zinc (black) showing the boundary between mMEC and PaS. **(D)** Same section processed for calbindin (green) and AChE (black) showing the boundaries of mMEC. Scale bars: 250 μm. D, dorsal; V, ventral; M, medial; L, lateral; R, rostral; C, caudal. Orientation in **(A)** also applies to **(C,D)**.

**FIGURE 3:**
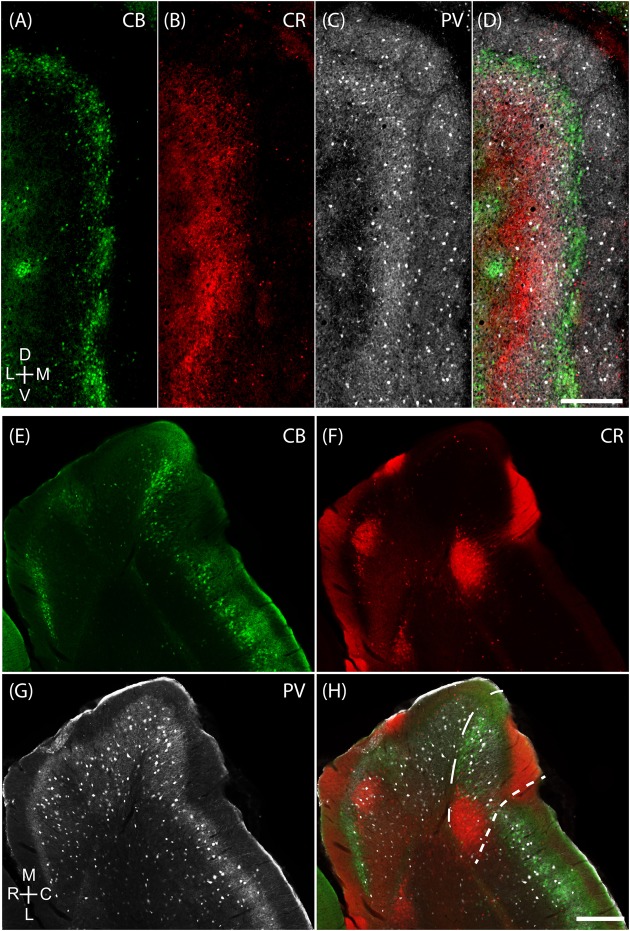
**Medial-most part of the medial entorhinal cortex layout**. The mMEC shows an organization of parallel stripes in tangential sections near the surface (upper panels). This pattern comes about by sectioning, from medial to lateral, through by a patch of calretinin positive neuropil located above a group of calretinin positive neurons, then a region with predominantly parvalbumin positive neuropil and then a narrow stripe of calbindin patches at the border to the parasubiculum. The same tangential section processed for calbindin (green, **A**), calretinin (red, **B**) and parvalbumin (white, **C**) show that they form adjacent bands in the mMEC **(D)**. **(E–H)** The same horizontal section processed for calbindin (green, **E**), calretinin (red, **F**) and parvalbumin (white, **G**) shows that the mMEC **(H)** contains a lower density of calbindin positive neurons, a relatively thick layer of parvalbumin positive neuropil and a patch of calretinin positive neurons extending their dendrites to the surface of layer 1. Scale bars: 250 μm (**D** also for **A–C**), (**H** also for **E–G**). D, dorsal; V, ventral; M, medial; L, lateral; R, rostral; C, caudal. Orientation in **(A)** applies to upper four panels **(A–D)** and orientation in **(G)** applies lower four panels **(E–H)**.

Layer 2 of the rodent MEC also has calbindin patches (see below; Fujimaru and Kosaka, 1996; Ray et al., 2014) while the adult rodent PaS lacks calbindin expression (Ray and Brecht, 2016). We found that the border between parasubiculum and MEC is marked by a narrow band of cells expressing both calbindin and Wfs1 (**Figures 2A,B**) in contrast to cells in superficial layers of the parasubiculum which express only Wfs1. This calbindin stripe lies at the border of the intense staining for several markers such as AChE, zinc ions and cytochrome oxidase which have been classically used to define the lateral border of the parasubiculum (Mathisen and Blackstad, 1964; Slomianka, 1992; Burgalossi et al., 2011; Tang et al., 2016). This calbindin stripe thus appears to demarcate the medial border of a ‘transition region’ between the MEC and the parasubiculum; based on this histochemical and cytoarchitectonical evidence we propose that this transition region might be distinct from the neighboring PaS and MEC; we will hence refer to this structure as ‘medial-most part of the medial entorhinal cortex’ (mMEC). Unlike MEC, the mMEC is devoid of calbindin patches but displays a narrow stripe of calbindin-positive neurons at its dorsal and medial end (**Figures 2C,D**). This calbindin-stripe lies on the lateral border with a region reactive for zinc (**Figure 2C**) and AChE (**Figure 2D**), which has been a classical marker of the parasubiculum. The narrow patches of calbindin positive cells at the border to the parasubiculum correspond to a narrow band of patches in tangential sections (**Figures 2A,C,D**, **3A**). Another characteristic feature of superficial layers of the mMEC is the presence of calretinin positive neuropil at the medial border of the mMEC (**Figure 3B**) and a broad band of parvalbumin positive neuropil (**Figure 3C**). This results in adjacent, largely non-overlapping stripes of calretinin-positive, parvalbumin-positive, and calbindin-positive regions (**Figure 3D**). We explore the complex underlying laminar architecture by staining a complementary horizontal section for the same markers (**Figures 3E–H**). The medial border of the mMEC is formed by a narrow band of calbindin positive cells that extend almost to the surface of the cortex (**Figure 3E**). Layer 3 of the mMEC contains a dense cluster of calretinin positive cells (**Figure 3F**), unlike other parts of the MEC. The parvalbumin positive neuropil of the mMEC forms a wider band than in the MEC but comparable to the parasubiculum (**Figure 3G**). In combination, these immunohistochemical markers illustrate the distinct laminar and modular structure of the mMEC (**Figures 3D,H**).

### Cellular Structure of Layer 2 of Medial Entorhinal Cortex

**FIGURE 4:**
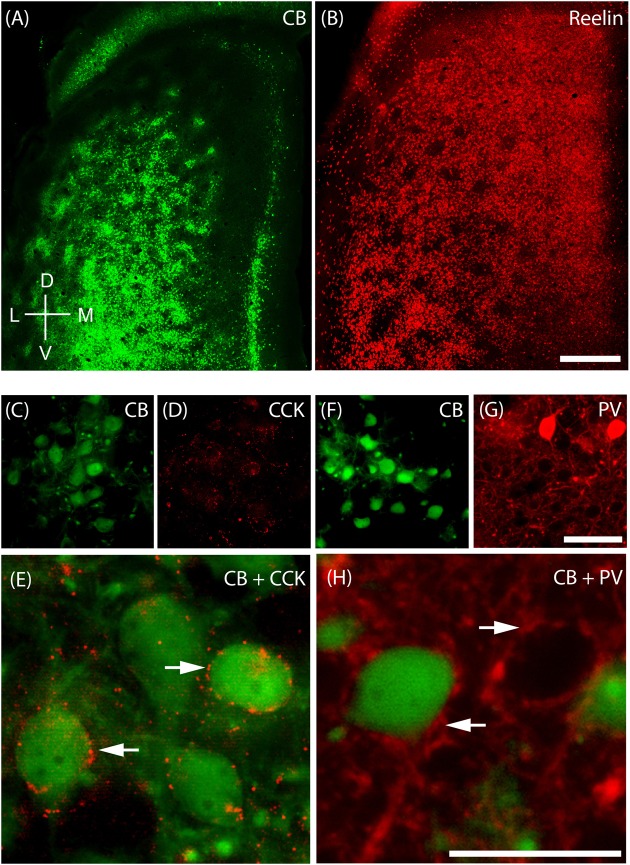
**Cellular structure of layer 2 of medial entorhinal cortex**. Calbindin (CB) and reelin-positive cells differ in spatial distribution and innervation by basket cells. **(A)** Tangential section through upper layer 2 of MEC stained for calbindin immunoreactivity. CB-positive neurons cluster and form characteristic patches. **(B)** In contrast reelin positive cells have a more uniform distribution and avoid the center of calbindin patches. **(C–E)** Cholecystokinin (CCK) positive terminals preferentially target calbindin-positive cells. Tangential section stained for CB **(C)** and CCK **(D)** immunoreactivity; enlarged overlay view with CCK positive terminals indicated by arrows in **(E)**. **(F–H)** Parvalbumin (PV) positive terminals target calbindin-positive and calbindin-negative cells. Tangential section stained for CB **(F)** and PV **(G)** immunoreactivity; enlarged overlay view **(H)** with PV-positive terminals indicated by arrows. Scale bars: **B** = 500 μm (also for **A**); **G** = 50 μm (also for **C,D,F**). **H** = 25 μm (also for **E**). D, dorsal; V, ventral; L, lateral; M, medial. Orientation in **(A)** also applicable to **(B)**.

The MEC contains two major principal cell classes, calbindin-positive pyramidal cells (**Figure 4A**) which cluster in patches and reelin-positive stellate cells, which are densely packed and mostly evenly spread out, but avoid the center of these calbindin patches in the superficial part of layer 2 (**Figure 4B**). We also find that CCK immunoreactivity in MEC is preferentially concentrated around CB patches (**Figures 4C,D**; Köhler, 1986a) and that CCK-positive puncta form baskets surrounding CB-positive cells (**Figure 4E**), in line with previous findings (Varga et al., 2010). In contrast, parvalbumin (PV) inputs seem to be rather indiscriminate in layer 2 (**Figures 4F,G**) with PV-positive puncta forming baskets around both CB-positive pyramidal cells and Reelin-positive stellate cells (**Figure 4H**; Armstrong et al., 2016).

### Zinc Modules are Complementary to Calbindin Patches, and Receive Largely Segregated Inputs from the Presubiculum and Parasubiculum Respectively

**FIGURE 5:**
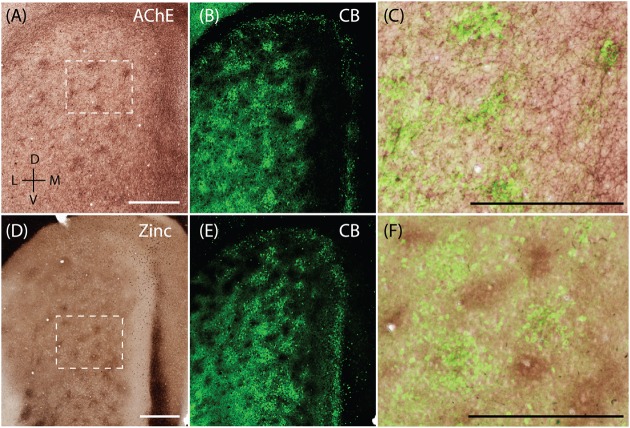
**Complementary modular systems in MEC**. Relation of acetylcholinesterase (AChE) and zinc-positive modules to calbindin patches. **(A)** Tangential section stained for AChE, showing discrete clusters of staining. **(B)** Same section as in **(A)** co-stained for calbindin, showing calbindin patches. **(C)** High magnification view of overlay from **(A,B)** showing that calbindin patches overlap with AChE innervation. **(D)** Tangential section stained for synaptic zinc ions, showing discrete clusters of staining. **(E)** Same section as in **(D)** co-stained for calbindin, showing calbindin patches. **(F)** High magnification view of overlay from **(D,E)** showing that zinc and calbindin form complementary modules. Scale bars: **A,C,D,F** = 500 μm (**A** also for **B, D** also for **E**). D, dorsal; V, ventral; L, lateral; M, medial. Orientation in **(A)** applicable to all. © 2014 The American Association for the Advancement of Science, **(A–C)** is adapted from Ray et al. (2014). Reprinted with permission from AAAS.

Calbindin patches in the rat MEC have been shown to receive preferential cholinergic innervation (Ray et al., 2014) and thus hotspots of AChE activity are co-localized with calbindin patches (**Figures 5A–C**, adapted from Ray et al., 2014). When staining for synaptic zinc, we also found a modular organization in the MEC (**Figure 5D**) and wondered if they also co-localized with the CB patches. The staining intensity of synaptic zinc increases slowly from dorsal to ventral in MEC and strongly in the transition to lateral entorhinal cortex (**Figure 5D**; Haug, 1973). Staining the same section for synaptic zinc and calbindin immunoreactivity (**Figures 5D,E**) reveals that calbindin patches and zinc modules are largely non-overlapping (**Figure 5F**) and form complementary modules in layer 2 of the MEC.

**FIGURE 6:**
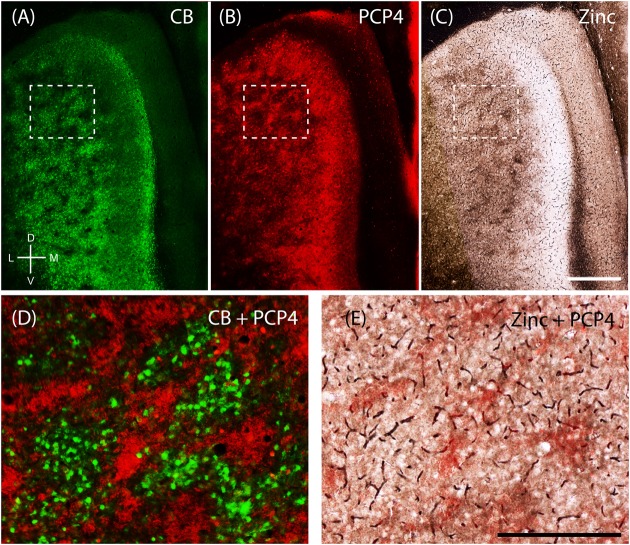
**Purkinje cell protein 4 (PCP4) is complementary to calbindin patches and overlaps with zinc modules**. Apical dendrites from layers 3 and 5 Purkinje cell protein 4-positive cells (PCP4) bundle together in layer 2. Same section of layer 2 of the entorhinal cortex processed for CB **(A)**, PCP4 **(B)** immunoreactivity and synaptic zinc ions **(C)**. **(D,E)** Inset from **(A–C)** showing that PCP4 is complementary to CB-patches **(D)** and overlaps with zinc-modules. Scale bars: **C** = 500 μm (also for **A,B**); **E** = 250 μm (also for **D**). D, dorsal; V, ventral; L, lateral; M, medial. Orientation in **(A)** applicable to all.

The apical dendrites of PCP4-positive cells from layers 3 and 5 of MEC bundle in MEC layer 2 (Tang et al., 2015). Thus, we tested whether PCP4-positive apical dendrites co-localize with the zinc modules. Indeed, processing the same section for CB (**Figure 6A**) and PCP4 (**Figure 6B**) immunoreactivity revealed that they form complementary modules (**Figure 6D**). Co-staining for pre-synaptic zinc (**Figure 6C**) demonstrates that the PCP4-positive dendritic bundles co-localize with the zinc modules (**Figure 6E**). Thus in the MEC, calbindin patches overlap with cholinergic inputs, while the complementary PCP4 clusters overlap with zincergic inputs. Notably, mMEC shows a rather high expression of PCP4 (**Figure 6B**) but distinctly lacks zincergic inputs (**Figure 6C**), further supporting our hypothesis that the mMEC shows distinct features to the neighboring MEC and PaS.

**FIGURE 7:**
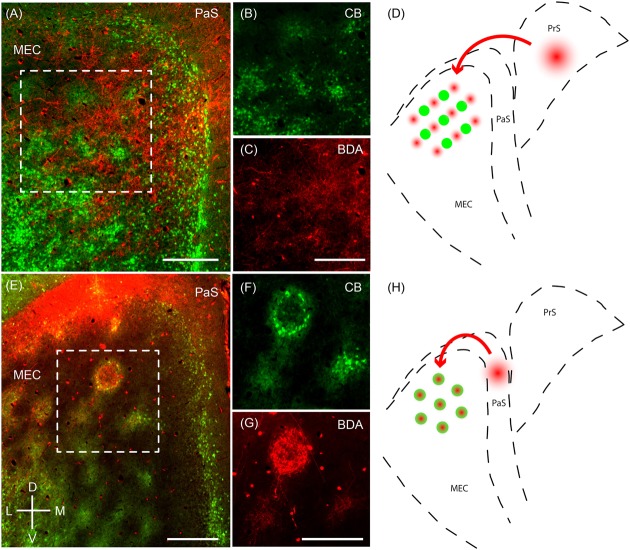
**Parasubiculum provides inputs to calbindin patches while presubiculum provides inputs to complementary modules**. Anterograde injections performed in the PaS and PrS target complementary modules in layer 2 of the MEC. **(A)** Tangential section of layer 2 of the MEC showing that axons of neurons from the presubiculum (red) largely avoid the CB patches (green). **(B,C)** Inset region from **(A)** showing calbindin patches **(B)** and complementary regions innervated by axons **(C)** from a BDA anterograde injection in the presubiculum. **(D)** Schematic illustrating that presubiculum neurons target modules complementary to the CB patches in layer 2 of MEC. **(E–G)** Tangential section illustrating an anterograde injection (BDA, red) performed in the parasubiculum showing selective targeting of CB patches (green) in layer 2 of MEC **(F,G)**. **(H)** Schematic illustrating that the parasubiculum targets CB patches in layer 2 of the MEC. Scale bars: **A,C,E,G** = 250 μm (**C** also for **B, G** also for **F**). D, dorsal; V, ventral; L, lateral; M, medial. Orientation in **(E)** applicable to all. **(E–G)** Adapted from Tang et al. (2016).

Next, we sought to resolve whether the two modular systems are embedded in distinct microcircuitry. To this end, we performed anterograde injections in the presubiculum and parasubiculum. Anterograde injections in the presubiculum targeted more densely the regions complementary to the calbindin patches (**Figures 7A–D**) – which correspond to the zinc/PCP4 modules (**Figures 5F**, **6D**). On the other hand, previous work has indicated that parasubiculum inputs are largely restricted to the calbindin patches (**Figures 7E–H**; adapted from Tang et al., 2016). This anatomical evidence therefore indicates that presubiculum and parasubiculum inputs to MEC target complementary modules in layer 2 of the MEC, and thus point toward parallel modular processing within L2 of MEC.

### Modularity in Pre and Parasubiculum

Modular organization is a defining feature of several parahippocampal areas (Wyss et al., 1990; Fujise et al., 1995). While we illustrated the complementary modules in L2 of MEC, here below we will explore more in detail the cytoarchitectonic organization their input structures, the PrS and the PaS.

**FIGURE 8:**
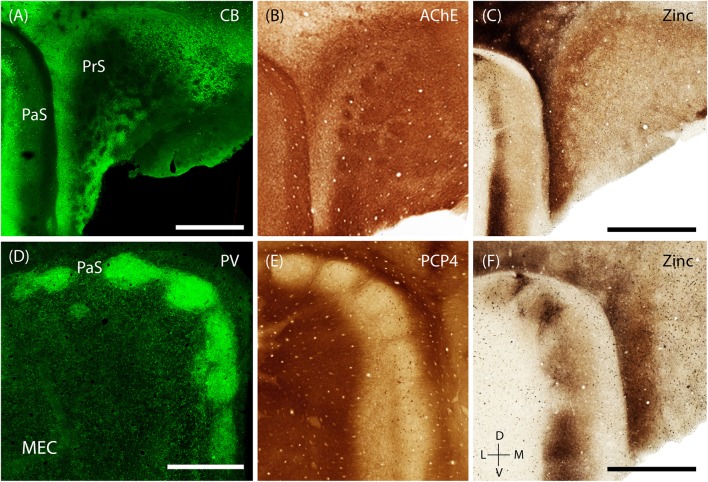
**Modularity of presubiculum and parasubiculum**. The pre and parasubiculum provides inputs to the zinc modules and calbindin patches, respectively, and also have a modular layout themselves. **(A–C)** Tangential sections showing modules in the presubiculum, with the modules being devoid of CB (green, **A**) and synaptic zinc **(C)** but being enriched for cholinergic activity **(B)**. **(D–F)** Tangential sections showing the presence of large modules in the parasubiculum, enriched for PV (green, **D**) and synaptic zinc **(F)** but lacking PCP4 **(E)**. Scale bars: **A,C,D,F** = 1 mm (**C** also for **B, D** also for **E**). D, dorsal; V, ventral; L, lateral; M, medial. Orientation in **(E)** applicable to all. **(D)** Adapted from Tang et al. (2016).

The presubiculum contains clusters, which are largely restricted to PrS L2, are surrounded by calbindin- positive cells (**Figure 8A**; Fujise et al., 1995; Preston-Ferrer et al., 2016). These clusters also stain for acetylcholinesterase (**Figure 8B**). Staining for synaptic zinc illustrates that the presubicular clusters lack zincergic inputs which are more enriched in the septae surrounding the presubicular L2 clusters (**Figure 8C**).

The PaS has also been shown to have a prominent modular organization (Burgalossi et al., 2011; Tang et al., 2016). The discrete structure of parasubicular patches is evident in superficial sections stained for cytochrome oxidase and myelin, the latter of which highlights the septae of the patches (Burgalossi et al., 2011). Staining for PV (a marker known to be particularly enriched in the PaS) shows that PV-positive cells and neuropil are enriched within the large parasubicular patches (**Figure 8D**, adapted from Tang et al., 2016) while PCP4-positive neuropil marks the septae surrounding the patches (**Figure 8E**). In contrast to the presubiculum, zincergic inputs are enriched in the parasubicular patches (**Figure 8F**).

## Discussion

The parahippocamal network consists of a host of reciprocally connected areas including the entorhinal cortex, the presubiculum and the parasubiculum. Due to the reciprocity of the connections, it’s often hard to determine which area generates a function and which area is involved in relaying or refining it. Understanding the functional anatomy of this region thus requires integrating information at the level of cell types, microcircuits, modular structures, and interareal connectivity (see Witter and Moser, 2006; Van Strien et al., 2009; Burgalossi and Brecht, 2014; Igarashi, 2016 for review). The MEC can be delineated by its columnar staining for AChE activity in layer 3 as opposed to the more homogeneous staining pattern seen across superficial layers in lateral entorhinal cortex. Previous work has shown that layer 2 of MEC consists of two principal cell classes, which can be differentiated according to calbindin and reelin immunoreactivity (Varga et al., 2010; Kitamura et al., 2014; Ray et al., 2014). These cells are receive different inhibitory inputs, with Varga et al. (2010) showing that cholecystokinin (CCK)-positive basket cell terminals specifically innervate calbindin- positive cells using VGluT3 as an indirect marker (Somogyi et al., 2004).

In the present study, by using histochemical, anatomical and tract-tracing techniques, we have further explored the modular organization of the MEC. Previous descriptions of modular distribution of histochemical markers in superficial layers of MEC of rodents include clusters of calbindin-positive cells (Fujimaru and Kosaka, 1996), zinc-positive terminals (Slomianka and Geneser, 1997) and acetylcholinesterase patches (Slomianka and Geneser, 1991), yet the interrelation of these different markers was unknown. Here we show that, in addition to the recently described calbindin-patches which overlap with AChE activity (Ray et al., 2014), the MEC contains a complementary modular system, involving dendrites of PCP4-positive L3 and L5 pyramidal cells overlapping with zinc activity (**Figure 6**; Tang et al., 2015). Interestingly, presynaptic zinc and PCP4 seem to have overlapping or complementary distribution throughout the entire hippocampal region (Ishihara and Fukuda, 2016). Previous work has indicated that zinc containing neurons are almost exclusively glutamatergic (Frederickson et al., 2000) and virtually all of the histochemically reactive zinc in the brain is present as presynaptic vesicular zinc (Danscher et al., 1985), which might act as a modulator of glutamate release (Takeda et al., 2003).

We also provided evidence that the two complementary modular systems in MEC L2, the calbindin patches and zinc modules, receive largely segregated inputs from the parasubiculum and presubiculum, respectively. Anterograde injections (**Figure 7**) in the parasubiculum revealed selective projections to the calbindin-patches in layer 2 of the MEC (Tang et al., 2016), while injections in the presubiculum terminated in the areas between the calbindin-patches, which correspond to the complementary zinc-modules. This is in line with Köhler’s interpretation (Köhler, 1985) where he traced inputs from the presubiculum and parasubiculum to the MEC and found them to terminate in clusters. Projections from presubiculum preferentially label axons in layer 3 and deep layer 1 of MEC and form thin septa in layer 2 MEC, while projections from parasubiculum terminate in relatively large clusters in MEC layer 2 (Witter et al., 1989; van Groen and Wyss, 1990). This is also supported at the single cell level by reconstructions of single axons from the presubiculum (Preston-Ferrer et al., 2016) and the parasubiculum (Burgalossi et al., 2011) to the superficial layers of the MEC. The connectivity from the presubiculum has been studied in detail by Witter and colleagues who showed that presubicular inputs target apical dendrites of layer 5 entorhinal neurons and also neurons in all other layers of entorhinal cortex (Caballero-Bleda and Witter, 1993; Wouterlood et al., 2004; Canto et al., 2012). Based on their correlated appearance in development, Slomianka and Geneser (1997) proposed that presubicular axons have zinc-positive terminals in the MEC, in line with our findings (**Figures 7A,D**). Taken together, we illustrate that in layer 2 of MEC, the parasubicular afferents preferentially target the calbindin patches, whereas presubicular afferents preferentially project to the space in between them, corresponding to the zinc modules. Physiologically, it has also been indicated that the properties of parasubicular cells are similar to the cells in the calbindin-patches (Ebbesen et al., 2016). It would be interesting to investigate if such a similarity also exists between presubicular cells and those in the zinc modules.

### Possible Functional Implications of the Complementary Modules

Calbindin patches form periodic clusters in a number of mammalian species (Naumann et al., 2016) and represent a rather evolutionarily conserved unit. The patches are arranged in a hexagonal pattern in rats, which seems to be a consequence of genetic patterning (Ray and Brecht, 2016) since they are present before the appearance of functional grid cells (Langston et al., 2010; Ray and Brecht, 2016). However, whether the functional periodicity of the grid cells is shaped by the anatomical layout (Brecht et al., 2013) or is independent of it, is still an open question. Several studies have so far explored the cellular basis of the grid representation. At first, due to their direct projections to the dentate gyrus, stellate cells were considered to be the prime source of grid cell activity. Later studies have however, proposed that pyramidal cells might as well-contribute to the grid representation (Tang et al., 2014; Sun et al., 2015). In the latter studies, grid cells represented only a fraction of all neurons (Tang et al., 2014; Sun et al., 2015), thus indicating that further subdivisions are likely to exist within the broad principal cell classes (Fuchs et al., 2016). In light of the present findings, it would be interesting to resolve whether and how grid cells are related to the two complementary modular systems in MEC L2 and the largely segregated inputs they receive from the presubiculum and parasubiculum. Both the pre- and parasubiculum contain a large proportion of head direction cells (Boccara et al., 2010; Tang et al., 2016) which have been proposed to play a crucial role in the generation of grid activity. Head-direction cells in both PaS (Burgalossi et al., 2011) and PrS (Preston-Ferrer et al., 2016) have been shown to project to the MEC, and they might represent two parallel sources of HD inputs to grid cell system in MEC.

### Complementary Modules and Alzheimer’s Disease

The entorhinal cortex is one of the first areas affected in Alzheimer’s disease and both AChE and zinc have been implicated in its onset and progression. Patients suffering from Alzheimer’s disease show a marked reduction in AChE activity (Davies and Maloney, 1976; Coyle et al., 1983) and the use of drugs boosting cholinergic function can ameliorate memory deficits (Davis et al., 1979). Similarly, zinc plays a role in Alzheimer’s disease with aggregates of zinc co-localizing with amyloid-beta plaques in Alzheimer’s patients (Lovell et al., 1998). However, different types of cells in rodents (pyramidal) and humans (stellates) show increased AChE activity (Naumann et al., 2016), and it would also be interesting to investigate how the distribution of zinc in humans relates to rodents. Understanding of these divergent evolutionary principles would also enable us to better translate cell-type specific results from rodents to humans.

### *mMEC:* The Medial-Most Part of the Medial Entorhinal Cortex

The region at the medial extremity of the MEC is devoid of calbindin patches and zinc modules and exhibits a rather different structure than the rest of the MEC. It has historically been described as either a part of the parasubiculum (Lorente de Nó, 1933, 1934) or the medial entorhinal (Blackstad, 1956), as a transitional zone (Chronister and White, 1975) or as a separate entity (Fujise et al., 1995; Fujimaru and Kosaka, 1996). We retained its name as the mMEC, following the nomenclature used by Fujimaru and Kosaka (1996). In the human parahippocampal region, a similar layout has been observed, with the region corresponding to the MEC displaying calbindin patches (Naumann et al., 2016) but the transition region between MEC and PaS being seemingly devoid of patches (Sgonina, 1938).

In the mMEC, we found adjacent dorso-ventral stripes of calretinin at the border with MEC (Miettinen et al., 1997), followed by parvalbumin positive neuropil and a narrow stripe of calbindin-positive cells forming the border with the PaS. The parvalbumin cells in the superficial layers of this region are exclusively GABAergic (Wouterlood et al., 1995). The majority of calretinin neurons were initially thought to be GABAergic but several reports found also calretinin positive glutamatergic neurons (Miettinen et al., 1997; Wouterlood et al., 2000, 2007, 2008). Hence, based on the overall distribution of GABAergic neurons in the parahippocampal region, it is likely that the densely clustered calretinin positive neurons in the mMEC are glutamatergic (Miettinen et al., 1997), but their transmitter identity remains to be ascertained.

Further studies, particularly connectivity studies, would be needed to ascertain, whether the mMEC should be considered a sub-region of the MEC or PaS, a transitional region between the two or a different region in its own right. It also remains to be conclusively ascertained whether the stripe of calbindin-positive cells at the border between mMEC and PaS should be considered as part of the mMEC or PaS. Ontogenetically, the PaS shows calbindin-expression in early development (Ray and Brecht, 2016), and by maturation only a narrow stripe remains at the border. However, this calbindin-stripe partially lies outside the traditional boundaries of the PaS, as determined by AChE and Zinc histochemistry. The mMEC also has a distinctive layout compared to the rest of the MEC, as determined by the distribution of calretinin, calbindin, and parvalbumin positive cells (**Figure 3**). Future functional, ontogenetic, and evolutionary studies would help us decipher the role of this region.

### Overview of Modular Structures in the Parahippocampal Region

Cholinergic and zincergic modules are not exclusive to the MEC. In several adjacent areas such as retrosplenial granular cortex (Ichinohe, 2012) and presubiculum, acetylcholinesterase staining shows a strikingly modular distribution, but at present it is not known how they relate to modular terminations of cholinergic fibers in the entorhinal cortex or elsewhere. The modular structure of the presubiculum is most prominent in primates and humans and was described in classical studies of cellular architecture (Rose, 1927; Altschul, 1933; Braak, 1978). More recently, detailed studies using chemoarchitecture, molecular markers and single-axon tracing have revealed the modular archictecture of the presubiculum in macaque monkeys in great detail (Ding et al., 2000; Ding and Rockland, 2001; Ding, 2013). Studies on the modular structure of the rodent presubiculum have focused on the development of cellular modules (Nishikawa et al., 2002) and have shown cytochrome oxidase modules in adult animals (Gonzalez-Lima and Cada, 1998) but are still relatively scarce. Integrating data on parahippocampal structure from rodents, primate, and other mammals is critical for translational research (Ding, 2013; Naumann et al., 2016), however, here we narrowly focus on the rat parahippocampal regions. Staining for acetylcholinesterase activity in retrosplenial granular cortex and presubiculum reveals distinct clusters as in MEC but also reveal a number of differences: (i) Each area has a distinct density and distribution of acetylcholinesterase activity clusters. (ii) MEC does not show a matching M2 receptor distribution (data not shown; Rouse and Levey, 1996; Wang et al., 2011). (iii) Calbindin-positive cells in presubiculum form a lattice surrounding acetylcholinesterase activity clusters, whereas there are few calbindin-positive cells in superficial layers of retrosplenial granular cortex.

**FIGURE 9:**
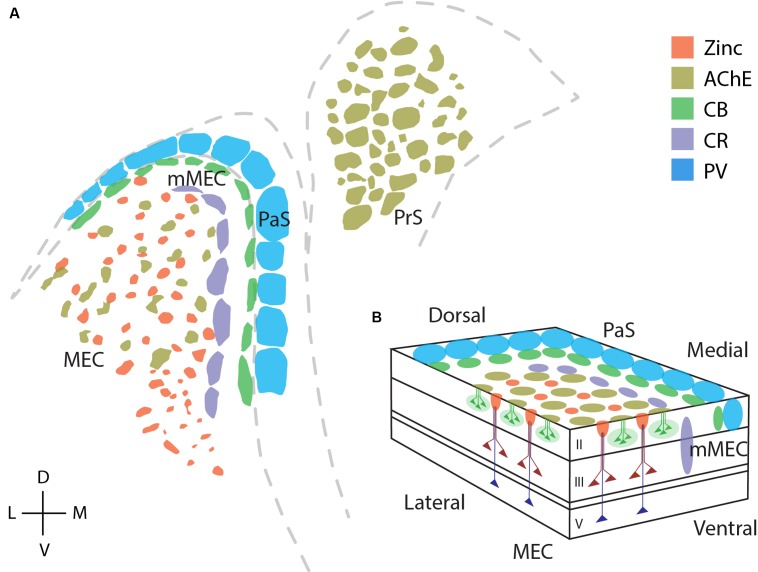
**Modular structures in the parahippocampal region**. Overview of modular structures in the superficial layers of MEC and neighboring regions. **(A)** Overview of modular structures in the superficial layers of MEC and the neighboring, mMEC, PaS, and PrS. Sections stained for different markers were aligned at the entorhinal/parasubicular border and modular structures drawn. **(B)** Schematic illustration of modular structures in the dorsal MEC and parasubiculum. D, dorsal; V, ventral; L, lateral; M, medial. © 2006 Elsevier, **(B)** adapted from Witter and Moser (2006). Adapted with permission from Elsevier.

**Figure 9** reviews the areal and modular organization of superficial layers of the entorhinal cortex and adjacent cortical areas, specifically modular structures in MEC, mMEC, parasubiculum and presubiculum (**Figure 9A**). **Figure 9B** shows an idealized scheme of the modular architecture of MEC and the adjacent mMEC and parasubiculum, adapted from Figure 3 of Witter and Moser (2006). Modular structures in layers 1 and 2 are depicted in a simplified way on the surface and the sides of layer 2. Calretinin cells form translaminar clusters with the highest density of cells in layer 3 (Miettinen et al., 1997). Pyramidal cells in layers 3 and 5 are displayed as in Figure 3 of Witter and Moser (2006); they form dendritic bundles, which terminate in between calbindin patches (Hamam et al., 2000; van Haeften et al., 2003; Wouterlood et al., 2004; Witter and Moser, 2006; Tang et al., 2015).

In summary, our evidence indicates the presence of two parallel information pathways in the parahippocampal region, which engage complementary modular systems in the MEC.

## Author Contributions

All authors had full access to all the data in the study and take responsibility for the integrity of the data and the accuracy of the data analysis. Study concept and design: RN, SR, MB. Acquisition of data: RN, SR, AB, MB. Analysis and interpretation of data: RN, SR, AB, MB. Drafting of the manuscript: SR, RN, MB. Critical revision of the manuscript for important intellectual content: SR, RN, AB, MB. Statistical analysis: SR, RN, MB. Obtained funding: MB, AB. Administrative, technical, and material support: AB, MB. Study supervision: MB.

## Conflict of Interest Statement

The authors declare that the research was conducted in the absence of any commercial or financial relationships that could be construed as a potential conflict of interest.
